# Worldwide paleodistribution of capillariid parasites: Paleoparasitology, current status of phylogeny and taxonomic perspectives

**DOI:** 10.1371/journal.pone.0216150

**Published:** 2019-04-30

**Authors:** Victor Hugo Borba, José Roberto Machado-Silva, Matthieu Le Bailly, Alena Mayo Iñiguez

**Affiliations:** 1 Laboratório de Helmintologia Romero Lascasas Porto, Faculdade de Ciências Médicas, Universidade do Estado do Rio de Janeiro (FCM/UERJ), Rio de Janeiro, Rio de Janeiro, Brazil; 2 Laboratório de Biologia de Tripanosomatídeos, Instituto Oswaldo Cruz, Fundação Oswaldo Cruz (IOC/FIOCRUZ), Rio de Janeiro, Rio de Janeiro, Brazil; 3 University of Bourgogne Franche-Comte, CNRS UMR 6249 Chrono-environment, Besançon, France; Onderstepoort Veterinary Institute, SOUTH AFRICA

## Abstract

**Introduction:**

Paleoparasitology, the study of parasites in the past, brings the knowledge of where and when they occurred in preterit populations. Some groups of parasites, as capillariids, have a complex and controversial systematic, hindering the paleoparasitological diagnosis. In this article, we synthesized the occurrence of capillariids in both the New and the Old World in ancient times, and discussed the difficulty of the diagnosis of species and the strategies for identification. The present review also shows the current status of the phylogeny in capillariids and indicates the necessity to try new approaches for a better understanding of capillariid paleodistribution.

**Methods:**

For the systematic review, a predefined guideline defined by PRISMA was used. The articles collected were identified, screened, and included in the review following criteria for eligibility. The current status of the phylogeny of capillariids was accessed using MUSCLE, Bioedit v.7.0.5 and MEGA v. 7.0.21 programs.

**Results:**

The review discussed 38 articles that presented information about capillariids in past populations. Most of capillariid eggs found in the New and Old World were not identified. However, *Calodium hepaticum* eggs were the most identified, as some from *Eucoleus* genus. It was observed that sites from the New World had a better chance for capillariid egg identification, due to previous knowledge of its host, when compared to the Old World. In the 18S rDNA phylogenetic analyses, two datasets were constructed, one including sequences from 7 Moravec’s genera, where 3 genus-specific clusters, with high bootstrap values, could be observed for *Capillaria* (ML = 99%, NJ = 96%), *Eucoleus* (ML / NJ = 100%) and *Paratrichosoma* (ML / NJ = 100%). A fourth cluster of 18S rDNA dataset I revealed lack of definition of *Pearsonema* and *Aonchotheca* genera. The 18S rDNA dataset II comprised 8 Moravec’s genera and defined 3 clusters, 2 genus-specific for *Eucoleus* (ML = 99%, NJ = 100%) and *Capillaria* (ML / NJ = 98%). The third 18S rDNA dataset II cluster included 6 genera and exhibited, once again, *Pearsonema* and *Aonchotheca* poor discrimination. The *cox*1 gene data consist of 4 Moravec’s genera, and in spite of grouping some species-specific clusters, did not show genera-specific definition.

**Conclusions:**

Despite the numerous archaeological findings, both in the New and the Old Worlds, the identification of capillariid species based on the morphology and morphometry of eggs remains imprecise, often resulting in a generic diagnosis of a group or morphotype of capillariid. Capillariid is one of the most diverse group of helminths recovered in archaeological sites. The phylogenetic trees produced in this study showed limited genetic information available, unresolved genera and incongruence with the classical taxonomy. The elucidation of the paleodistribution of capillariids can give insights of the ancient host-parasite associations but also in modern sceneries.

## Introduction

Over 300 species of capillariids have been described throughout the world in different vertebrate hosts, as fishes, amphibians, reptiles, birds or mammals [1]. The difficulty of identification, due to the small size of the specimens and the few morphological structures that are characteristics for species identification, make their systematic and taxonomy one of the most complex among the phylum Nematoda. Another factor that hinders their classification is the phenotypic plasticity caused by different hosts, infection sites and locations [1]. In 1982, Moravec changed the classification of the group when proposed 16 genus of capillariids [2]. After that, a number of new genera, synonyms and reclassifications have been proposed.

Currently, it is know that capillariids are spread throughout the world, with a slight knowledge of the genus or species distribution within the different hosts and localities. However, the scenario of the paleodistribution of capillariid species in the past is very uncertain. There are a few findings, both in the New and in the Old World, in a sort of archeological material, such as coprolites, sediment associated to skeleton, latrines, pits and cesspits, with uncertain diagnoses of species. Most of taxonomic classification in the papers is *Capillaria* sp., which does not necessary means the species identification of *Capillaria* genus, as proposed by Moravec (1982). In other publications, the capillariid genus is specified.

There are scarce molecular and phylogenetic studies regarding capillariids, most of them focus on 18S rDNA or *cox*1 targets, considering just a few of the current known genera. Some authors showed an integrative study, which gives a robust molecular result of species discrimination. Despite the little information, it was possible to discriminate some genera as *Eucoleus* or *Capillaria* [3].

In this study, we present and discuss the paleoparasitological findings and paleodistribution of capillariids worldwide. In addition, we present the current status of the phylogeny and suggests new taxonomic perspectives for the understanding of the capillariid paleodistribution.

## Materials and methods

**Fig 1:**
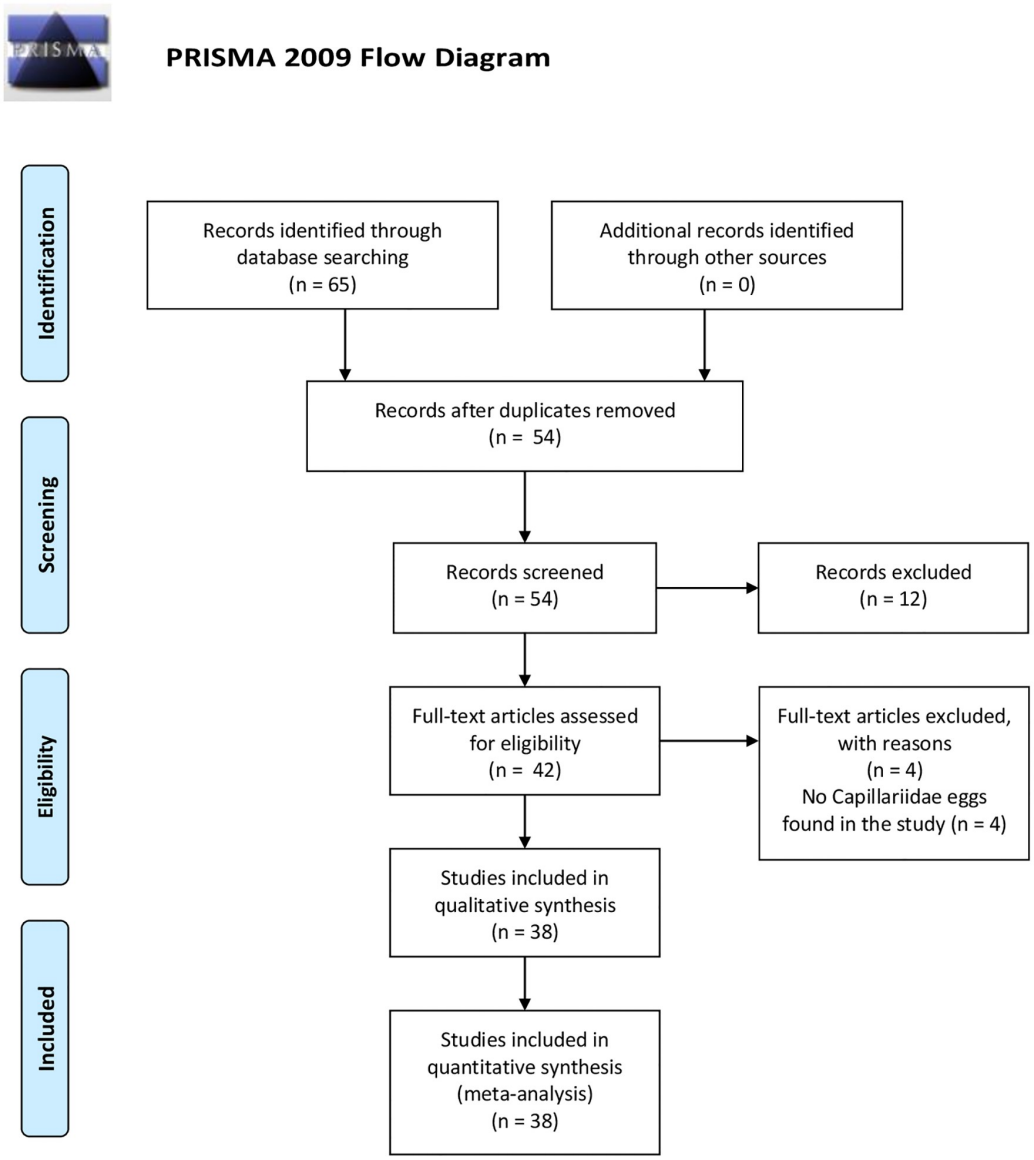
PRISMA flow diagram. From: Moher D, Liberati A, Tetzlaff J, Altman DG, The PRISMA Group (2009). Preferred Reporting Items for Systematic Reviews and Meta-Analyses: The PRISMA Statement. PLoS Med 6(7): e1000097. doi:10.1371/joumal.pmed1000097 **For more information, visit** www.prisma-statement.org.

In this systematic review we followed a guideline defined by PRISMA [4]. This process generated a PRISMA flow chart illustrated in Fig 1. Basically, the literature was identified as possible articles, then screened and accesses for eligibility and included in the review.

### Paleoparasitological literature compilation

The literature review aimed to identify and summarize the findings of capillariid eggs on archaeological samples in any context, and to show its paleodistribution. A database was made by articles collected on PubMed and ScienceDirect using keywords such as (“capillariid” OR “Capillariidae” OR “Capillariinae” OR “*capillaria*” or “Trichuridae”) AND (“paleoparasitology” OR “archaeoparasitology” OR “coprolite” OR “ancient samples”).

In spite of the *Capillaria* sp. diagnosis in some studies, in this review, we particularly revised the efforts conducted by some authors to reach a taxonomic identification of *Capillaria* genus, otherwise we used the generic nomenclature “capillariids”, which involves all genera including *Capillaria* sp.

### Capillariid phylogenetic analysis

Phylogenetic analyses were conducted to establish the current genetic relationships among the species to elucidate its complex taxonomy and phylogenetic arrangement to corroborate with the taxonomic classification of Moravec [2].

All sequences available in GenBank (08/2018) of the 18S rDNA and *cox*1 mtDNA genes were used for the phylogenetic analysis. Sequences with reduced sequence size were not used, as 18S rDNA from *Paracapillaria philippinensis* (235 bp). Sequence edition alignment and visualization were performed in Bioedit v.7.0.5 and MUSCLE [5]. Genetic distance matrixes for each genus / species group were constructed to determine the inter and intraspecific distances between genus and species using the Kimura-2-parameter with Gamma distribution (K2P + G) on MEGA v. 7.0.26 [3]. In the 18S rDNA gene, since most of sequences were located at the 5’ or the 3’extremities, the alignment was separated in two datasets to use the largest number of genus / species sequences available. The names of the species were maintained as was deposited in GenBank, but the genus names were specified according to the taxonomic classification proposed by Moravec [2]. Identical sequences or with genetic distances less than 0.020 were excluded.

Dataset I included 105 sequences (1090 bp) of 16 species belonging to 7 genera of capillariids: *Pearsonema*, *Aonchoteca*, *Baruscapillaria*, *Pseudocapillaria*, *Paratrichosoma*, *Eucoleus*, and *Capillaria*. In addition, sequences of undefined genus were included. *Trichuris* and *Trichinella* species sequences were used as outgroup. Dataset II was of 40 sequences (678 bp) of 18 species belonging to 8 genera: *Pearsonema*, *Aonchoteca*, *Baruscapillaria*, *Pseudocapillaria*, *Pseudocapillaroides*, *Eucoleus*, *Capillaria*, and *Calodium*. The outgroup was the same as in the previous analysis.

For the phylogenetic tree construction, two methods were applied in MEGA [3]. Neighbor-Joining (NJ) and K2P + G model following the protocol for the molecular identification of species (DNA barcoding CBOL protocol) (http://www.barcodeoflife.org/content/resources/Standards-and-guidelines), and Maximum Likelihood (ML), also using the K2P + G model, as determined by the best-fit model of DNA substitution command using the Bayesian information criteria in MEGA. In both, the statistical support of the branches was generated by 500 bootstrap replicas.

A Dataset for the *cox*1 gene consisted of 30 GenBank sequences (255 bp) of 7 species belonging to 4 genera: *Pearsonema*, *Aonchoteca*, *Eucoleus*, and *Calodium*. *Trichuris* and *Trichinella* species sequences were used as outgroup. The NJ method was based on the K2P + G model as advocated by the barcoding analysis and the ML method was based on the Tamura 3-parameter model. Both with Gamma distribution and supported by 500 bootstrap replicas.

## Results

### Paleodistribution of capillariids

#### Capillariids in the New World

**Fig 2:**
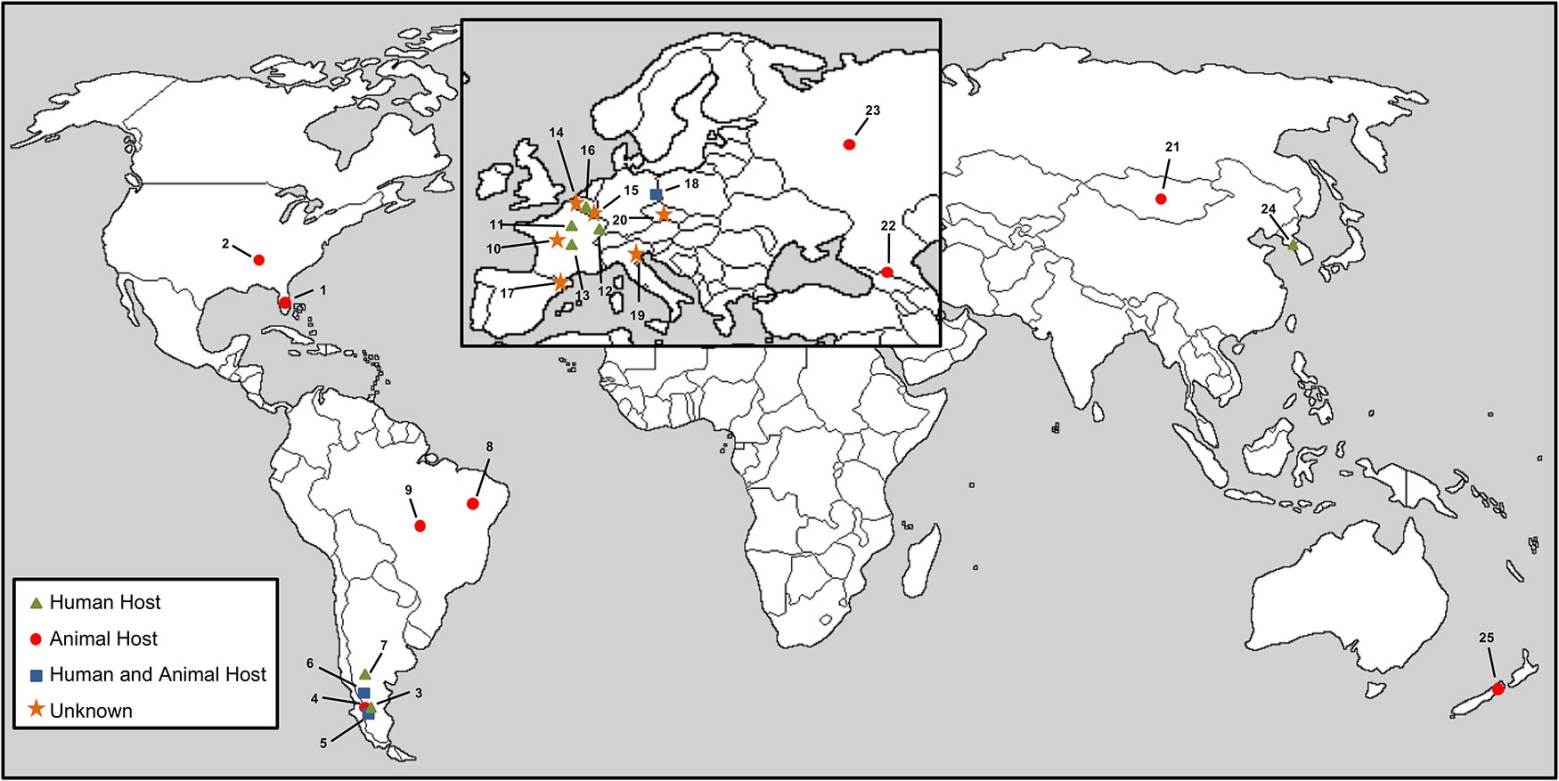
Worldwide paleodistribution of capillariids. The symbols represent the locals where the parasites were found in the archeological material. Relative data see map numbers in Tables 1 and 2. The host of capillariids is indicated whenever is known.

Capillariid findings in the New World are not much numerous than in the Old World (Fig 2). In North America, only two studies have identified capillariid eggs. Reinhard and coauthors (1986) were the first to find capillariid in archaeological material, i.e. samples of crocodile coprolites dated about 6000 years before present (BP), collected in the United States, in the Florida state. It was possible to observe the presence of two species of capillariid in which despite the good preservation of the eggs, the larval material had been degraded [6]. McConnell and Zavada (2013) found capillariids in sediments associated with skeleton of an extinct tapir (*Tapirus polkensis*), which lived in the Miocene-Pliocene period (4.5–7 million years BP). The authors revealed very poorly preserved structures that have similar characteristics and measures to capillariid eggs [7]. This structure may represent an evidence of a longstanding host-parasite interaction of capillariid with this group of mammals.

In South America, the research on archaeological material, mostly coprolites, have revealed numerous findings of capillariid eggs. It is noteworthy the findings in Patagonia region, Argentina. There are a number of studies with the identification of different morphotypes of capillariid eggs in a range of hosts, such as canids, rodents, camelids, felids, birds and even humans.

The first publication, by Fugassa and Guichón, (2005), showing capillariid parasites in archaeological material from Patagonia, was in human coprolites [8]. In the same region, sediments taken from skeletons and graves dating from 3720–3978 BP were also analyzed. In this material, capillariid eggs were identified, but the authors suggested the presence of more than one species, since a variety of measures and ornaments on the eggshells was found [9]. Two studies examined parasites in sediment associated with sacral region. The first study was conducted on skeletons recovered in three archaeological sites (Nombre de Jesús, Las Mandíbulas, Caleta Falsa) from the Patagonia region, and stored in museum collections in Argentina. Although the cleanse of the skeletal remains, as part of museological curation process, the recovery of helminth eggs was possible with the identification of two capillariid morphotypes, one of them attributed to *Calodium hepaticum* [10]. In the second study, an egg of capillariid was found in a rodent coprolite found in sediment associated with a body dating from the Hispanic post-contact period (212 ± 35 years BP) [11].

Some coprolites of uncertain zoological origin, with characteristics of both human and canid feces, were also analyzed in the Patagonia region. Coprolites dated of 3480 and 2740 years BP, showed eggs of *Calodium* sp. (n = 98), and another unidentified capillariid species (n = 4) [12]. In coprolites with older dating (9730 ± 100 to 8920 ± 200 years BP), corresponding to the transition from the Pleistocene to Holocene, the presence of *Calodium* sp. eggs suggested the consumption by hunter-gatherer of infected rodents, possibly by *C*. *hepaticum* [13]. The taxonomic identification was performed based on eggshell ornaments, which have particular striations that permit the genus discrimination. A canid coprolite (6540 ± 110 years BP), probably from *Pseudalopex culpaeus*, revealed numerous eggs (n = 171) with different measures, that were identified as capillariid [14].

Feline coprolites were also analyzed in Patagonia [15]. A number of *Calodium* sp. eggs (n = 563) were found and identified as *C*. *hepaticum* based on the morphometric data and the similarity of eggshell ornaments. Taking into consideration the life cycle of the parasite, the finding could represent an environment contaminated by *C*. *hepaticum*, which could have affected humans who shared the same location. In the same study, another capillariid, similar to *Eucoleus* sp. was also found, but in lower frequency (n = 4) [15]. A carnivore coprolite from Epullán Chica site, in Argentina, revealed one egg of *Eucoleus* sp., probably a *Eucoleus aerophilus* [16].

Although the morphological characteristics of *C*. *hepaticum* egg are very singular (small average size and typical ornamentation), the possibility of other species with similar egg morphology cannot be ruled out. The analysis of a coprolite of camelid from Patagonian region, dating 8300 ± 130 to 7920 ± 115 BP [17], revealed eggs similar to *C*. *hepaticum*. However, the infection is not supported by the parasite life cycle and their herbivore host diet. Therefore, it was suggested that other species with eggs similar to *C*. *hepaticum* circulated in that environment. Coprolites of camelid collected from the Andean region, dated to 9640 ± 190 to 3920 ± 80, revealed three capillariid morphotypes. One morphotype was compatible with *Calodium* sp., which usually infects the gastrointestinal tract of a variety of host, and has been found in coprolites of humans, canids, felids and rodents. Other morphotype was comparable to *Eucoleus* sp. and the third morphotype had not matching with any capillariid described in camelids until that time [18]. An study done in this region showed once more the same morphotype compatible with *Calodium* sp. and one other morphotype never seen before in camelid coprolites, increasing the number of species found in this host [19].

In coprolites of rodents, dating from 7920 ± 130 years BP, the presence of capillariids identified as *Eucoleus* sp. (n = 3), and other helminth eggs were described. Although the genus identified represent parasites that usually infect the respiratory tract, the presence in coprolites could be a result of swallowing of expelled secretion with eggs [20]. Another analysis in rodent coprolites showed a high number (n = 239) and variety of capillariid morphotypes (n = 4). The eggs were identified as suggestive of genera *Calodium*, *Eucoleus*, *Echinocoleus* and an unidentified capillariid species [21].

Besides coprolites, ancient pellets were analyzed, which are structures resulting of the regurgitation process of prey birds. Some studies have performed microscopic analysis on this material and showed capillariid eggs with radially ornamented eggshell, and morphometry compatible with *C*. *hepaticum*. Because rodent bones, often preys of raptors, were recovered in the pellets, Fugassa and collaborators (2007) attributed the infection to rodents [22]. In Beltrame and coauthors (2011), despite the identification of *C*. *hepaticum* eggs in raptor pellets, it was pondered the presence of others capillariid species attributed to the genera *Aonchotheca* and *Eucoleus*, which include some species that parasitize bird esophagi [23]. The archaeological site has several caves and rock shelters with evidence of human occupation, suggesting a probable exposure of humans to zoonotic parasites of rodents and, perhaps, of birds [23].

In Brazil, only two findings of capillariids are known in coprolites. Sianto and coauthors [24] recovered *Calodium* sp. eggs (N = 2) in feline coprolite (2840 ± 100 years BP), collected in Serra da Capivara, Northeast Brazil. Morphologically identified as *Calodium* cf. *hepaticum*, the finding could represent a case of false parasitism by consuming the parasite’s final host, and consequently, the eggs appeared in feces without acquiring the infection [24]. Confalonieri [25] identified *Echinocoleus hydrochoery* (syn. *Capillaria hydrochoery*) in coprolites of capybara (*Hydrochoerus hydrochoeris*) from Lapa da Angélica archaeological site, Goiás state, central west region. Currently, the parasite affects capybaras in the region. However, the coprolite eggs are slightly larger (Table 1) than those found nowadays [25].

**Table 1:** Capillariids on New World.

Locality/Country	Period (BP)	Sample	Host	Capillariid identification	Measures (μm)	Map number	References
Florida / USA	6000–7000	Coprolite	Alligator	Capillariid morph1	-	1	[6]
				Capillariid morph2	-		
Tennessee / USA	4.5–7 m.a.	Sediments	*Tapirus polkensis*	Capillariid	54 x 30	2	[7]
CCP5 / Santa Cruz / Argentina	6540±110	Coprolite	Human	Capillariid	-	3	[8]
CCP/ Santa Cruz / Argentina	6540±110	Coprolite	Canid	Capillariid	27.5–85 x 20–47.5 (n = 174)	4	[14]
CCP/ Santa Cruz / Argentina	2740	Coprolite	Human or Canid	*Calodium* sp.	47.5–77 x 30–42.5 (n = 87)	5	[12]
				Capillariid	55–70 x 28.8–43 (n = 4)		
	3480			*Calodium* sp.	53–75 x 33.5–42 (n = 11)		
CCP5 / Santa Cruz / Argentina	6540±110	Coprolite	Feline	*Calodium hepaticum*	57.5–75 x 35–45 (n = 563)	4	[15]
				*Eucoleus* sp.	67.5 x 35.7 (n = 4)		
CCP7 / Santa Cruz / Argentina	7920±130	Coprolite	Rodent	*Eucoleus* sp.	60–62.5 x 37.5–40 (n = 3)	4	[20]
CCP/ Santa Cruz / Argentina	3440±70–6700±70	Coprolite	Rodent	*Calodium* sp.	60–70 x 33.7–47.5 (n = 153)	4	[21]
				*Eucoleus* sp.	50–55 x 22.5–35 (n = 56)		
				*Echinocoleus* sp.	65 x 31.5 (n = 1)		
				Capillarid morph	55–67.5 x 32.5–42.5 (n = 29)		
CCP7 / Santa Cruz / Argentina	9730±100–8920±200	Coprolite	Human or Canid	*Calodium* sp.	60–71.25 x 28.7–42.5 (n = 48)	5	[13]
CCP7 / Santa Cruz / Argentina	9640±190–3920±80	Coprolite	Camelid	*Calodium hepaticum*	55–70 x 32.5–45 (n = 47)	4	[18]
				*Eucoleus* sp.	80–87.5 x 45–52.5 (n = 5)		
				Morphotype	80–87.5 x 45–57.5 (n = 7)		
Patagonia / Argentina	Historic	Sediments*	Human	*Calodium hepaticum*	- (120–360 eggs/g)	3	[10]
	850			Capillarid morph	60 x 30 (n = 1)		
CCP5 / Santa Cruz / Argentina	6540±110	Pellet	Rodent	*Calodium hepaticum*	37.5–42.5 x 63.7–68.7 (n = 4)	4	[22]
CCP / Santa Cruz / Argentina	3990±80–2740±100	Pellet	Rodent	*Calodium* sp.	35–45 x 62.5–75 (n = 60)	4	[23]
OB1 / Santa Cruz/ Argentina	3575–3931	Sediment*	Human	Capillariid morph1	56–65 x 25–32.5	3	[9]
	3720–3978			Capillariid morph2	55.5–62.5 x 36.2–37.5		
				Capillariid morph3	62.5–72 x 35		
CCP7 / Santa Cruz / Argentina	8300±130–7920±115	Coprolite	Camelid	*Calodium hepaticum*	63.7–70 x 35–37.5	4	[17]
ADG / Santa Cruz / Argentina	3440±70–4900±70	Coprolite	Camelid	*Calodium sp*.	57.5–77.5 x 32.5–50 (n = 116)	4	[19]
				Capillariid	61.25–67.5 x 37.5–47.5 (n = 10)		
Rio Mayo / Chabut / Argentina	212±35	Coprolite	Rodent	Capillariid	65 x 35 (n = 1)	6	[11]
Epullán Chica / Patagonia / Argentina	2220±50	Coprolite	Carnivores	*Eucoleus aerophilus*	62.5 x 27.5 (n = 1)	7	[16]
Serra da Capivara / Piauí / Brazil	2840±100	Coprolite	Feline	*Calodium* cf. *hepaticum*	52.2–54 x 31.1–33 (n = 2)	8	[24]
Lapa da Angélica / Goiás / Brazil	-	Coprolite	Capybara	*Echinocoleus hydrochoery*	49–41 x 24–19	9	[25]

*Sediment extracted from skeletal remains

BP, before present; CCP, Cerro Casa de Piedra; m.a., millions years ago; cf., confer; eggs/g, eggs per gram of feces; morph, morphotype; -, no information available in the paper.

The identification of eggs in all studies is defined as capillariids, although it could include all genera, or be restricted to *Capillaria* genus. The species identification is infrequent, due to morphological variety of capillariid eggs, an important lack of knowledge on egg structures, and/or by the action of taphonomic agents that would have altered the structure of eggs and therefore, made the taxonomic identification more difficult. It was possible to suggest the capillariid species in few cases (Table 1).

#### Capillariids in the Old World

As in the New World, capillariid findings in the Old World are quite frequent. However, unlike the New World, the findings are more common in sediments, latrine, or burial, mainly in Western Europe where most of the analyses were conducted (Table 2).

**Table 2:** Capillariids on Old World.

Locality/Country	Period (BP)	Sample	Host	Capillariid identification	Measures (μm)	Map Number	References
Beauvais / France	Cen. 13 to 17	Latrine	-	Capillariid	-	10	[26]
Jura / France	3200–2980	Coprolite	Human	Capillariid morph1	70 x 31.5	11	[27]
				Capillariid morph2	62 x 35		
Carspach / France	1915/16 (First World War)	Sediment	Human	*Eucoleus gastricus*	65.5±1.6 x 28.6±0.4 (n = 6)	12	[28]
Amiens / France	Cen. 3 to 4 (Roman)	Burry	Human	*Calodium hepaticum*	46.7–25	13	[29]
Raversijde / Belgian	Cen. 16	Sediment	-	Capillariid	-	14	[30]
Place d’Armes / Namur / Belgian	Cen. 2 and 3 (Gallo-Roman)	Latrines and Pits	-	Capillariid	-	15	[31]
	Cen. 9–11 (Carolinian)			Capillariid	24 x 50		
	Cen. 12 and 13			Capillariid	-		
Nivelles / Belgian	Cen. 10–13 (Medieval)	Sediment	Human	Capillariid	- (n = 332)	16	[32]
La Draga / Lake Banyoles / Spain	7270–6930	Sediment	Soil	Capillariid morph1		17	[33]
				Capillariid morph2			
Saale-Unstrut Valley / Germany	4500	Sediment	Human and Cattle	Capillariid	-	18	[34]
EmiliaRomagna / Italy	Cen. 10–11 (Medieval)	Pits	-	Capillariid	- (n = 1913)	19	[35,36]
Prague / CzechRepublic	Cen. 18 and 19	Pits and Cesspits	-	Capillariid	33 x 15	20	[37]
Shahr-e Sukhteh / Iran	5150–3750	Coprolite	Sheep	*Aonchotheca bovis*	47.5–59.8 x 27.5–35.5 (n = 3)		[38]
Mongolia	1440	Coprolite	Rodent	Capillariid	-	21	[39]
North Ossetia	700	Coprolite	Goat	Capillariid	-	22	[40]
Moscow / Russia	Neolithic and Mesolithic	Coprolite	Canid	Capillariid	-	23	[40]
Korea	Cen. 17 (JoseonDynasty)	Mummy	Human	*Paracapillaria philippinensis*	34–35 x 17–20	24	[41]
New Zealand	<3000 and 6268±31	Coprolite	Moa	Capillariid	52–60 x 30–35 (n = 1423)	25	[42]

BP, before present; Cen., century or centuries; morph, morphotype; -, no information described in the paper.

In France, Bouchet [26] accomplished the first recovery of capillariid egg in the Old World. Sediments of latrines and pits from a medieval site in Beauvais (13^th^ to 17^th^ century) revealed capillariid eggs [26]. Later, during the investigations on the lakeside archaeological site of Chalain (Jura, France), human coprolites dated to the Neolithic period (5216–4996 years BP) tested positive for two different morphotypes of capillariids. The first presenting reticulated outer eggshell, and the second presenting punctuated outer eggshell [27]. The same two morphotypes were recovered during the analysis of several Neolithic sites from Germany and Switzerland in Lakes Federsee and Bodensee [28].

In the scenario of World War I, Le Bailly et al. (2014) analyzed samples from German soldiers [29]. The samples were collected in the abdominal cavity of three bodies, recovered in a military gallery collapsed in March 1918. Two individuals showed capillariid eggs, which were identified using morphometric analysis as *Eucoleus gastricus*, a parasite of rodents. The authors explained the finding by the historical data recording the presence and the circulation of rats inside the galleries, as well as, the close contact and interaction between soldiers and these rodents.

An unusual capillariid finding was made in a body dating from the Roman period in France [30]. X-ray and cross section analysis identified two hydatid cysts, probably developed by the larval stage of *Echinococcus* sp. tapeworm. Analysis of the residue allowed to visualize *Calodium hepaticum* eggs, showing a coinfection by these two parasites [30].

In Belgium, in the Raversijde archaeological site (16^th^ century) mainly inhabited by fishermen, few eggs of capillariids were observed in organic materials [31]. Based on the findings of eggs of *Trichuris trichiura* and *Ascaris lumbricoides*, it was possible to suggest the human origin of the material. In the Place d’Armes site in Namur, which had seven stratigraphic layers representing different historical ages, organic materials were collected from latrines, pits and cesspits. Capillariid eggs were found in layers dated to the Roman period (2^nd^-3^rd^ centuries), the Carolinean period (9^th^-11^th^ centuries), and a medieval period from12^th^ to 13^th^ centuries [32]. In addition, during the analysis performed in the historical center of Nivelles, dated to the medieval period, human samples from pelvic region were analyzed. One individual revealed a high abundance of capillariid eggs (332 eggs per gram), but the species could not be identified [33].

In Spain, a Neolithic site La Draga, dated of 7270–6930 BP, showed the presence of two capillariid morphotypes with different ornamentations. Considering the characteristics of the eggs, especially the ornamentation, the authors suggested that the reticulated-type egg was from a Bovidae infection and the punctuated-type was probably related with fish-eating habits or the presence of rodents [34].

In Germany, Dittmar and Teegen (2003) found capillariid eggs in sediment associated with pelvic region of human and cattle, dated of 4500 years BP. The authors considered the finding as a probably contamination by feces of rodents [35].

In Italy, an study analyzed pits from both Roman and Medieval period. These pits, particularly in the medieval period, were used as garbage, and contained organic materials, wood, charcoal, pottery and various artifacts. Capillariid eggs (n = 1913) were found in two samples. Because of the nature of the samples it was not possible to associated the parasite to a host [36,37].

Finally in the Czech Republic, it was also possible to recover capillariid eggs from a well and a pit belong to 18^th^and 19^th^ centuries, but here again no species discrimination could be made [38].

In Asia, some findings of capillariid were recorded in different hosts and contexts. In coprolites of rodents (*Alticola* sp.) from the eastern Mongolia, dating from at least 1440 years BP [39]; in coprolites of goats (*Capra cylindricornis*) from the North Ossetia republic, Russian Federation, dating from 700 years BP; and in canid coprolites from sites on northern Moscow, recovered from layers dating from the Neolithic and Mesolithic periods. Regarding the findings in goats, the authors discussed that the defeat of tribes in the region by Mongolian troops, followed the establishment of settlements with breeding of herds of these animals nearby, and consequently, the increased frequency of these parasites [40]. In Iran two morphotypes of capillariids were found in sheep coprolites, and assumed as *Aonchotheca bovis* due the host [41]. In Korea, capillariid eggs with morphometry comparable to *Paracapillaria philippinensis*, were identified in a human burial from the Joseon Dynasty (17^th^ century). However, the authors affirmed that further investigations should be conducted to defined the diagnosis because of the low number of cases of intestinal capillariasis is known in the country [42].

In Oceania, New Zealand, some studies with coprolites of extinct birds known as Moa was performed [43,44]. Coprolites dated of <3000 and 6368 ± 31 years BP of four moa species from different regions were analyzed. Capillariid eggs were found in two moa species from different regions, but the parasite species could not be identified [43].

Despite the lack of findings in Africa, that could be explained by less paleoparasitological material studied in the region, capillariids are distributed worldwide (Fig 2). Both, animal and human capillariids were found in the New and the Old World since several millenniums, which show a presence and a circulation of zoonotic capillariids, as *C*. *hepaticum*, in ecosystems that were inhabited by humans and animals. Besides, humans as other carnivores can act as paratenic hosts, carrying and dispersing the parasite in the environment.

#### Morphological differentiation and taxonomic identification strategies

In attempt to identify capillariid eggs and understand the dynamics of the paleodistribution of different capillariid morphotypes, some works have performed biostatistical analysis.

First, Fugassa and collaborators (2008) based on data of findings in Patagonian archaeological material, conducted a statistical analysis to evaluate morphometric and morphological differences, considering the size of eggs, ornament patterns in eggshell and size of polar plugs. The results showed a significant discrimination between four egg morphotypes using the linear variables, length and width, with greater importance for width parameter. Thus, to the authors correlated egg morphotypes with some well-known species, such as *C*. *hepaticum*, *Aonchotheca putorii* and *E*. *aerophilus* [45].

The biometric analysis conducted by Taglioretti and coauthors (2014), evaluated length and width variables and investigated if eggs that had similar morphotype in different scenarios belong to the same species. In the analysis, the eggs were selected randomly from different archaeological samples and Permutation Multivariate Analysis of Variance (PERMANOVA) approach was applied. The result showed that hosts, diet, archaeological sites, or dating, had no significant effect in morphometry of capillariid eggs. Thus, the authors concluded that capillariid eggs with the same morphotype in different scenarios were, in fact, from the same species [46].

In samples from German soldiers of World War I, Le Bailly and collaborators (2014) applied a statistical approach for capillariid identification. The study used Gower Algorithm followed by a Multivariate Regression Tree on a data set on various capillariid eggs including measurements and ornamentation patterns. The statistical test suggested species definition of the egg as *E*. *gastricus*, and proving a complementary tool to conventional diagnostic techniques for taxonomic identification [29].

These studies showed that with a mathematical approach it might be possible to achieve the differentiation and identification of capillariid eggs found in archaeological material. This approach can supplement paleoparasitological data obtained by microscopy, molecular and immunological techniques.

### Current phylogenies of capillariids

**Fig 3:**
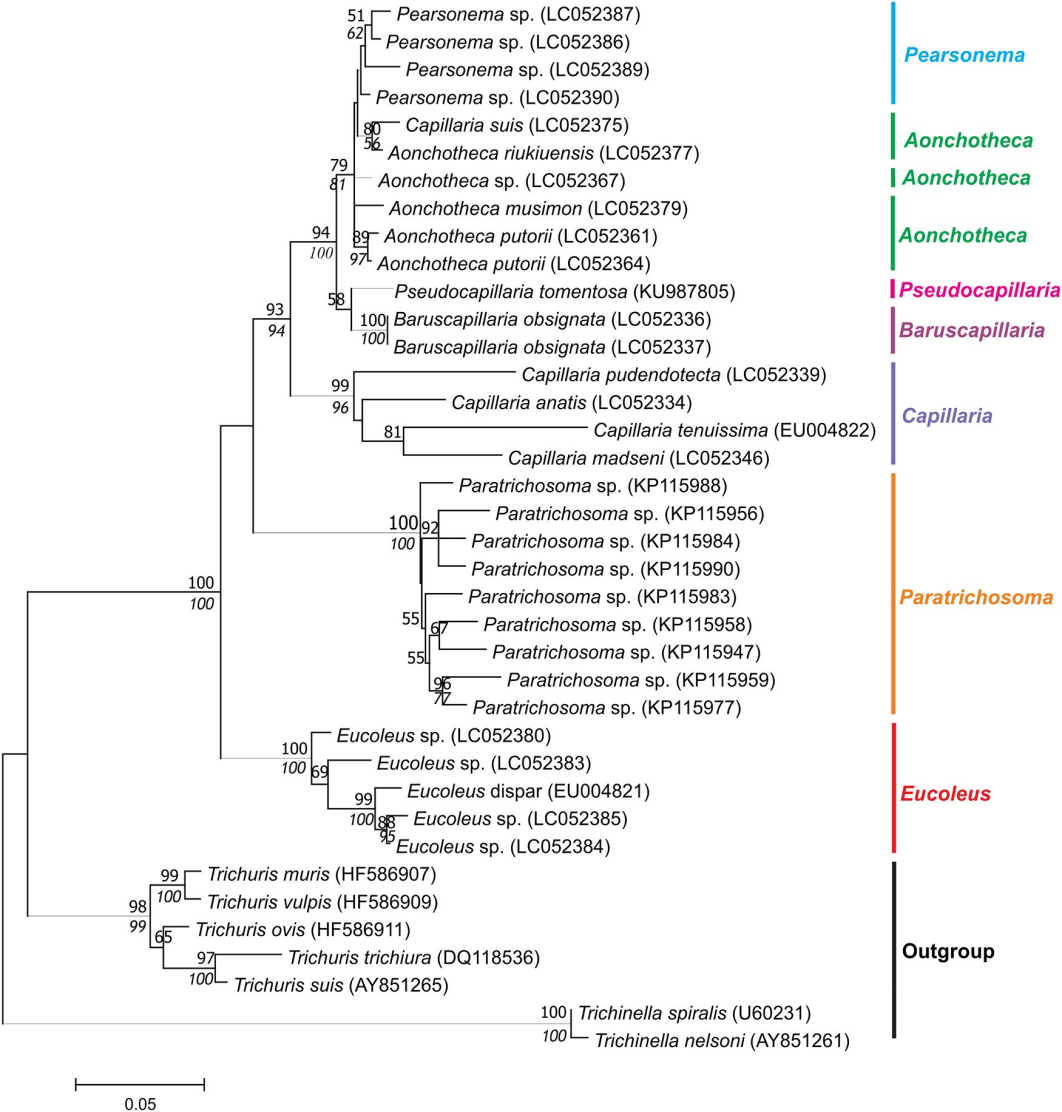
Dataset I phylogenetic tree based on 18s rDNA of capillariids inferred by MEGA v. 7.0.21 using Maximum Likelihood (ML) method, Kimura 2-parameter (K2P) model, and 500 replicates of bootstrap. Only bootstrap values ≥ 50% are shown. Neighbor Joining values are on italic.

The phylogenetic trees generated for dataset I showed four clusters, three of them genus-specific with high statistical support: *Capillaria* (ML = 99%, NJ = 96%), *Eucoleus* (ML / NJ = 100%) and *Paratrichosoma* (ML / NJ = 100%). The fourth cluster included sequences from at least 4 genera and presented short branches with medium to high support (ML = 94%, NJ = 100%), but no genus-specific subclusters are observed. A lack of monophyletic definition is clear among species from *Pearsonema* and *Aonchotheca* (Fig 3). This is also confirmed by the low distance values between *Aonchotheca* and *Pearsonema* (0.037 ± 0.004) in the genetic matrix (S2 Table). In the distance matrix, a great distance was observed in the genus *Capillaria* (0.098 ± 0.008). The 18S rDNA gene does not appear to be informative in order to discriminate these genera, at least for the segment of this Dataset I.

**Fig 4:**
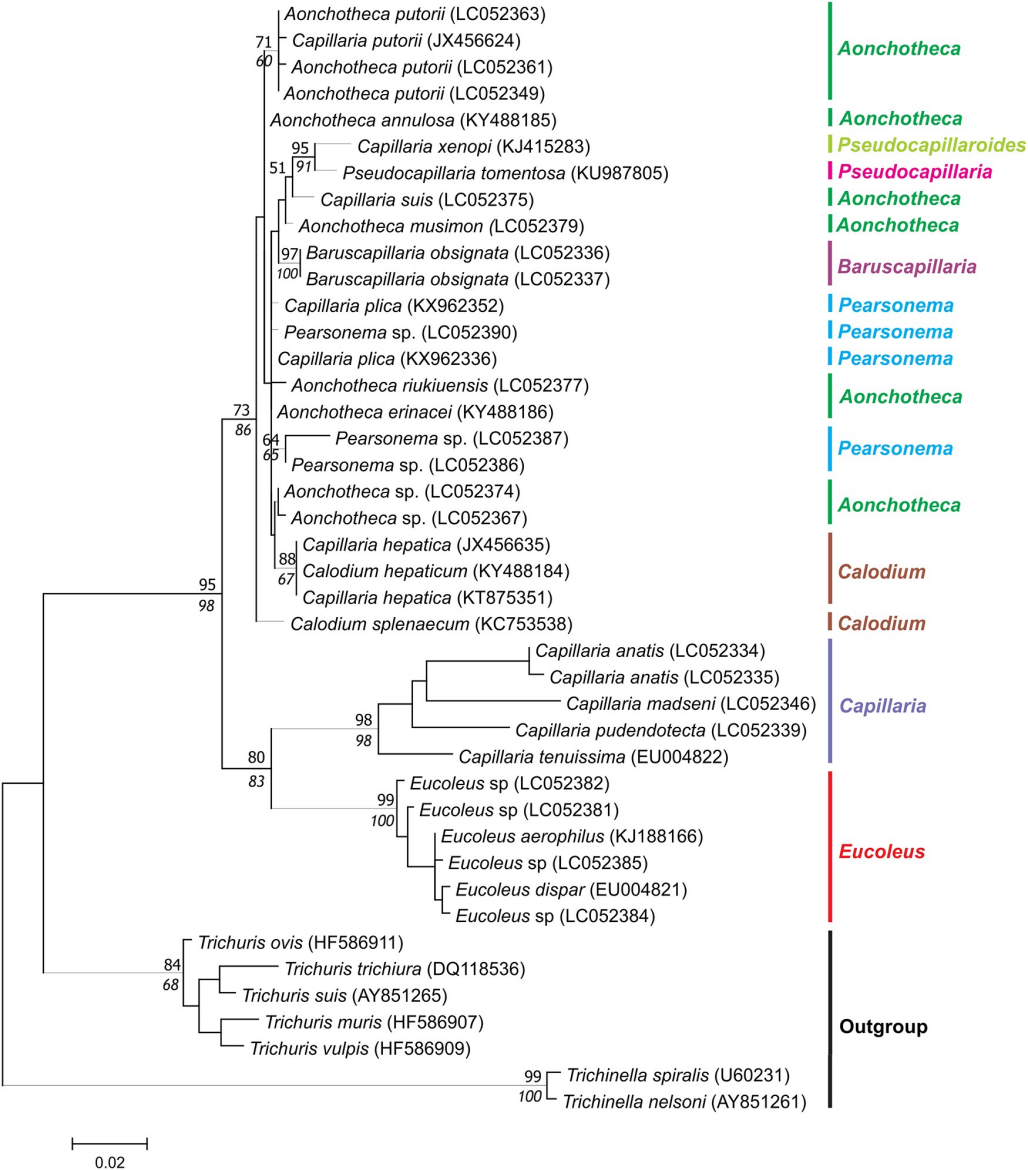
Dataset II phylogenetic tree based on 18s rDNA of capillariids inferred by MEGA v. 7.0.21 using Maximum Likelihood (ML) method, Kimura 2-parameter (K2P) model, and 500 replicates of bootstrap. Only bootstrap values ≥ 50% are shown. Neighbor Joining values are on italic.

In the trees generated with 18S rDNA dataset II (Fig 4), the strongly supported monophylies of the genera *Eucoleus* (ML = 99%, NJ = 100%) and *Capillaria* (ML /NJ = 98%) are also observed. A third cluster, with medium support (ML = 73%, NJ = 86%), grouping 6 genera, including 12 species, but not with monophyletic subclusters. *Calodium* spp. appear paraphyletic. *Calodium splenecum* species was basal, and *Calodium hepaticum* grouped with *Aonchotheca* spp. sequences. Once more *Pearsonema* spp. sequences clustered with *Aonchotheca* species despite their morphological differences.

In relation to the genetic analyses of Dataset II, the genus *Aonchotheca* presented one of the highest distances (0.027 ± 0.007) with *Pseudocapillaroides* and the shortest with *Aonchotheca* and *Pearsonema* (0.007 ± 0.002) within the cluster showing a paraphyletic origin. *Aonchotheca* and *Pearsonema* genera revealed an evolutionary distance (0.007 ± 0.002) smaller than between genera with similar posterior and spicule regions, such as *Baruscapillaria* and *Pearsonema* (0.015 ± 0.005), or *Aonchotheca* and *Calodium* (0.012 ± 0.004) (S3 Table).

The morphological characteristics of the posterior region in the adult male are taxonomic informative for the discrimination of capillariid genera [2]. The close proximity observed in our results could not be explained by Moravec taxonomic key based the hypothetical evolution of capillariids [2]. The author proposes that genera *Pearsonema* is evolutionary close related to *Baruscapillaria*, while *Aonchotheca* is close related to *Calodium*.

**Fig 5:**
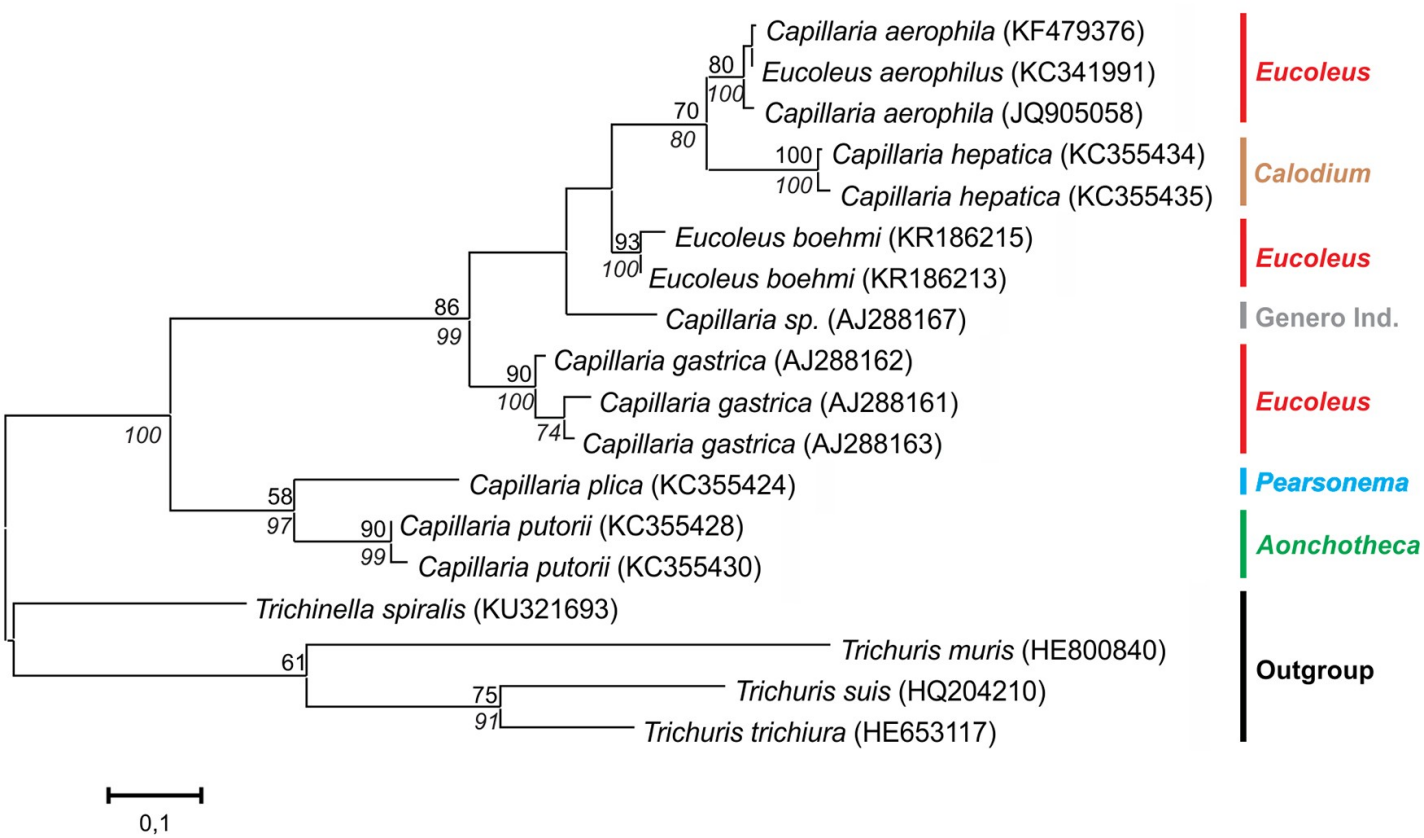
Phylogenetic tree based on *cox*1 gene of capillariids inferred by MEGA v7.0 using ML method, Tamura 3-parameter model, and 500 replicates of bootstrap. Only bootstrap values ≥ 50% are shown. Neighbor Joining values are on italic.

Unlike the 18S rDNA phylogeny, no genus-specific cluster was found in the *cox*1 phylogenetic trees generated (Fig 5). However, species-specific clusters were observed as noted before by Guardone et al. [47]. The genus *Pearsonema*, although represented by a single sequence, grouped with *Aonchotheca* for both models analyzed (ML = 58%, NJ = 97%). *Eucoleus* genus was paraphyletic, which was reflected by greater intraspecific distance (0.080 ± 0.012), and the smaller interspecific distance with the genus *Calodium* (0.161 ± 0.029). *Aonchotheca* and *Pearsonema* genera demonstrated a low interspecific distance (0.159 ± 0.031) (S4 Table), as also observed in the 18S rDNA analyses.

## Remarks on worldwide paleoparasitological findings of capillariids

Despite the numerous archaeological findings, both in the New and the Old Worlds, the identification of capillariid species based on the morphology and morphometry of eggs remains imprecise, often resulting in a generic diagnosis of a group of capillariid. The challenge in working with the characterization of egg morphology and morphometry is clearly due to the diversity of species and the similarity of eggs from different species. A coupled of statistic treatment have showed interesting results in the identification of taxon. However, this approach is limited by the poor knowledge on the egg morphology, mainly considering animal capillariids. Paleogenetic studies have been shown as promissory approach for identifying a variety of parasitic species in archeological materials [48–51]. Molecular techniques have not been employed in ancient capillariids and require an important development in the genetic characterization of the capillariid group.

**Fig 6:**
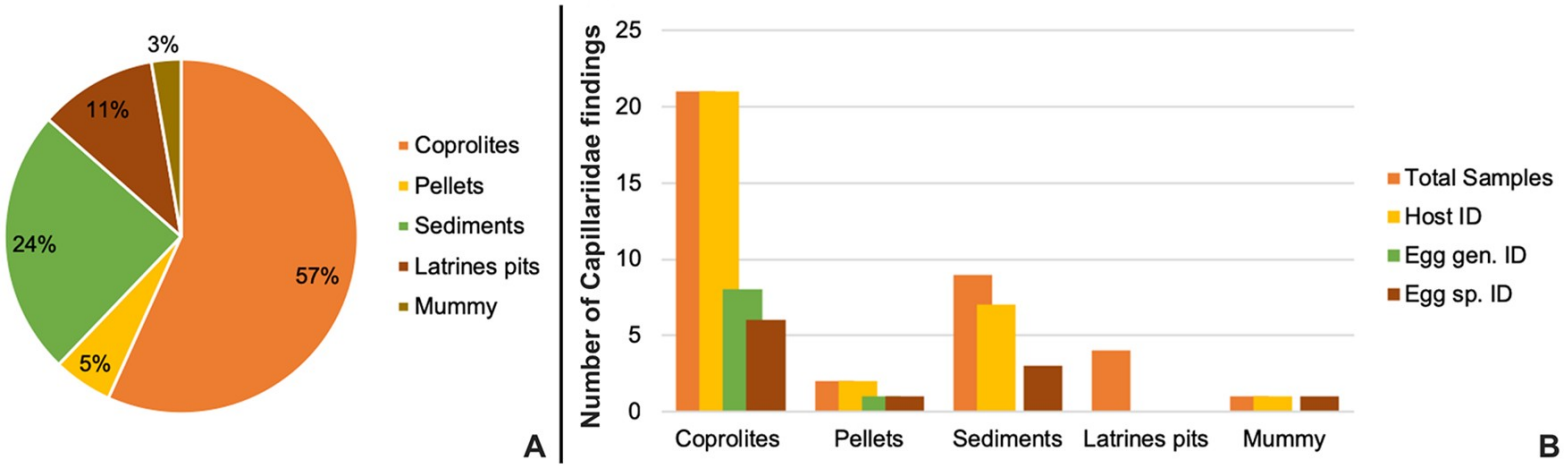
Graphical distributions of Capillaridae findings (n = 37) included in the present systematic review. (A) Distribution by types of samples; (B) Distribution by host and taxonomic egg identifications.

In this study, it was observed that most of samples were coprolites 21/37 (57%) (Fig 6A), which is an expected result since they are the main source of paleoparasitological studies. Characteristics of coprolites, including morphology, morphometry and vestiges, are informative of their origin. The review showed that all Capillaridae findings from coprolites had their host defined (21/21), therefore it was the type of sample with higher number of species and genera identified (14/21) when compared to other materials (6/16) (Fig 6B).

We summarized that from 54 egg morphotypes found in archaeological samples only 11 (20.37%) were identified at species level. Seven of them were recognized as *C*. *hepaticum*, the species with most singular morphological peculiarities. In other 6 morphotypes indicated as *Calodium* sp., it is possible to observe clear characteristics of *C*. *hepaticum*, but the species definition was not mentioned, which limited the biological, ecological and archaeological interpretations of results. From the ecological point of view, *C*. *hepaticum* eggs appear in human feces when liver and related viscera of the animal host are consumed. Consequently, this scenario does not represent a true infection, but show that the parasite, and their animal hosts, are circulating in the environment. In addition, the specific parasite identification could be understood as indirect archaeological evidence of lifestyle, diet and/or practices that involve a close relationship with a variety of hosts or a particular contact with site of infection.

In Le Bailly et al [29], the precise identification of *E*. *gastricus*, parasite of rodents, in WWI human samples, gives a possibility of a deeper interpretation of the host-parasite relationship, indicating a false parasitism related to soldier environments and practices. Eggs found in a Korean human mummy [42] could be identified as *P*. *philipinensis*, due to the associating of egg morphology and host origin. Regarding the parasite life cycle, the finding permits us to suggest a diet of the individual based on fish since it is the unique final host of *P*. *philipinensis*. In the same way, the identification of *C*. *hepaticum* eggs in feline coprolite by Sianto et al [24] would allow to suggest the animals involved in the network of transmission by integration of the data of parasite’s final host and the prey of felines that had habited Northeast Brazil.

Four morphotypes were identified at species level. In two, *E*. *aerophilus*, and *E*. *hydrochoery*, the information of the host made the parasite identification possible. The first is a carnivore parasite and the second a parasite specific of capybaras, respectively.

At genus level only 5 morphotypes were identified (Fig 6B), 4 as *Eucoleus* and 1 as *Echinocholeus*, both genera parasitize birds and mammals. The *Eucoleus* sp. morphotypes were found in feline and camelid, and both genera in rodents. The other 32 morphotypes were classified as family taxa, as the authors could not found taxonomic characters for a more specific definition, although 14 capillariids hosts were identified (Fig 6B). We remark that host data is the most value information for capillariid taxonomic identification (Fig 6B), after, obviously, the morphological and morphometric data. However, some capillariids have a large range of hosts, so little could be restricted from the broad species spectrum in the parasite definition.

As all samples studied from the New World had information about the parasite host, the egg identification was probably easier than the Old World parasites, since the host information is not available in most of samples. The importance of host origin data for the taxonomic discrimination results is ratified.

Despite the limited number of publications that conduct any statistical analysis of morphometric information for egg identification, the author effort applied with this objective, could be a bias. All studies that applied statistical evaluation of egg measures and structures reached an identification on genus or even species level [29,45,46].

The ornamentation of the wall of eggs is a very important character for taxonomic identification of capillariids, and we could notice that most of the studies did not described it properly, possible because the lack of specific pattern of nomenclature. Researches focusing the detailed differentiation of these eggs structure is crucial for a robust classification in future findings.

Molecular techniques have shown promising results in parasite diagnosis in archeological material [47,50], and new methodologies are rising facilitating the recovery of parasite aDNA [48,49]. The insertion and/or comparison of aDNA sequences with modern capillariid phylogeny could open discussions on taxonomy, host-parasite association or even on parasite evolution. Since the paleoepidemiology of capillariids is not truly known as not the properly species identification, studies involving molecular and morphological characterization of eggs, of as many capillariid species as possible, would help to show a most clear and complex picture of their distribution in the past and present. The phylogenetic trees produced in this study showed limited genetic information available, unresolved genera and incongruence with the classical taxonomy. It is evident the necessity of more genetic studies, manly of integrative taxonomy, in order to solve taxonomic conflicts, and to complement the systematic in Capillariidae, that, in addition, would permit the design of paleogenetic approaches. The elucidation of the paleodistribution of capillariids can give insights of the ancient host-parasite associations but also in modern sceneries.

## Supporting information

S1 Table PRISMA checklist. (DOCX)

S2 Table 18S rDNA– Dataset I. K2P Distance Matrix with estimates of evolutionary divergence over sequence pairs between groups. Bold numbers are those of evolutionary divergence within groups.
(DOCX)

S3 Table 18S rDNA– Dataset II. K2P Distance Matrix with estimates of evolutionary divergence over sequence pairs between groups. Bold numbers are those of evolutionary divergence within groups.
(DOCX)

S4 Table *cox*1 gene. K2P Distance Matrix with estimates of evolutionary divergence over sequence pairs between groups. Bold numbers are those of evolutionary divergence within groups.
(DOCX)
