# Supplementary material for: Worldwide paleodistribution of capillariid parasites: Paleoparasitology, current status of phylogeny and taxonomic perspectives
Source: PLoS One. 2019 Apr 30;14(4):e0216150. doi: 10.1371/journal.pone.0216150 (PMC6490956; doi:10.1371/journal.pone.0216150)
Supplement: S2 Table — K2P Distance Matrix with estimates of evolutionary divergence over sequence pairs between groups. Bold numbers are those of evolutionary divergence within groups. (DOCX) [file pone.0216150.s002.docx]

|  | 1 | 2 | 3 | 4 | 5 | 6 | 7 | 8 |
| --- | --- | --- | --- | --- | --- | --- | --- | --- |
| 1. Outgroup | **0.152** | *0.020* | *0.019* | *0.020* | *0.020* | *0.019* | *0.019* | *0.018* |
| 2. Paratrichosoma | 0.282 | **0.026** | *0.016* | *0.015* | *0.016* | *0.016* | *0.015* | *0.014* |
| 3. Capillaria | 0.268 | 0.209 | **0.098** | *0.014* | *0.011* | *0.012* | *0.010* | *0.010* |
| 4. Eucoleus | 0.253 | 0.185 | 0.174 | **0.053** | *0.012* | *0.012* | *0.011* | *0.011* |
| 5. Baruscapillaria | 0.254 | 0.178 | 0.130 | 0.130 | **0.010** | *0.008* | *0.008* | *0.008* |
| 6. Pseudocapillaria | 0.240 | 0.171 | 0.135 | 0.123 | 0.058 | **-** | *0.007* | *0.007* |
| 7. Pearsonema | 0.244 | 0.171 | 0.127 | 0.126 | 0.060 | 0.051 | **0.031** | *0.004* |
| 8. Aonchotheca | 0.243 | 0.165 | 0.123 | 0.119 | 0.059 | 0.047 | 0.037 | **0.023** |

**S1 Table. 18S rDNA – Dataset I**. K2P Distance Matrix with estimates of evolutionary divergence over sequence pairs between groups. Bold numbers are the of evolutionary divergence within groups.
