# Supplementary material for: Worldwide paleodistribution of capillariid parasites: Paleoparasitology, current status of phylogeny and taxonomic perspectives
Source: PLoS One. 2019 Apr 30;14(4):e0216150. doi: 10.1371/journal.pone.0216150 (PMC6490956; doi:10.1371/journal.pone.0216150)
Supplement: S3 Table — K2P Distance Matrix with estimates of evolutionary divergence over sequence pairs between groups. Bold numbers are those of evolutionary divergence within groups. (DOCX) [file pone.0216150.s003.docx]

|  | 1 | 2 | 3 | 4 | 5 | 6 | 7 | 8 | 9 |
| --- | --- | --- | --- | --- | --- | --- | --- | --- | --- |
| 1. Outgroup | **0.119** | *0.026* | *0.021* | *0.020* | *0.021* | *0.020* | *0.019* | *0.022* | *0.023* |
| 2. *Capillaria* | 0.162 | **0.045** | *0.016* | *0.014* | *0.013* | *0.014* | *0.014* | *0.015* | *0.016* |
| 3. *Pseudocapillaroides* | 0.126 | 0.089 | **-** | *0.007* | *0.007* | *0.007* | *0.007* | *0.015* | *0.005* |
| 4. *Pearsonema* | 0.117 | 0.074 | 0.025 | **0.003** | *0.005* | *0.003* | *0.002* | *0.013* | *0.006* |
| 5. *Baruscapillaria* | 0.125 | 0.072 | 0.024 | 0.015 | **0.000** | *0.005* | *0.005* | *0.013* | *0.007* |
| 6. *Calodium* | 0.120 | 0.077 | 0.025 | 0.009 | 0.018 | **0.006** | *0.004* | *0.013* | *0.007* |
| 7. *Aonchotheca* | 0.118 | 0.076 | 0.027 | 0.007 | 0.017 | 0.012 | **0.006** | *0.012* | *0.007* |
| 8. *Eucoleus* | 0.137 | 0.084 | 0.077 | 0.063 | 0.063 | 0.063 | 0.062 | **0.008** | *0.015* |
| 9. **Pseudocapillaria** | 0.134 | 0.085 | 0.017 | 0.021 | 0.024 | 0.024 | 0.024 | 0.074 | **-** |

**S2 Table. 18S rDNA– Dataset II**. K2P Distance Matrix with estimates of evolutionary divergence over sequence pairs between groups. Bold numbers are the of evolutionary divergence within groups.
