# Supplementary material for: Worldwide paleodistribution of capillariid parasites: Paleoparasitology, current status of phylogeny and taxonomic perspectives
Source: PLoS One. 2019 Apr 30;14(4):e0216150. doi: 10.1371/journal.pone.0216150 (PMC6490956; doi:10.1371/journal.pone.0216150)
Supplement: S4 Table — K2P Distance Matrix with estimates of evolutionary divergence over sequence pairs between groups. Bold numbers are those of evolutionary divergence within groups. (DOCX) [file pone.0216150.s004.docx]

|  | 1 | 2 | 3 | 4 | 5 | 6 |
| --- | --- | --- | --- | --- | --- | --- |
| 1. Outgroup | **0.386** | *0.058* | *0.049* | *0.045* | *0.050* | *0.063* |
| 2. *Pearsonema* | 0.423 | **0.003** | *0.031* | *0.046* | *0.057* | *0.052* |
| 3. *Aonchotheca* | 0.388 | 0.159 | **0.016** | *0.038* | *0.046* | *0.046* |
| 4. *Eucoleus* | 0.392 | 0.320 | 0.277 | **0.080** | *0.030* | *0.029* |
| 5. Undefined Genus | 0.398 | 0.350 | 0.277 | 0.175 | **0.007** | *0.042* |
| 6. *Calodium* | 0.485 | 0.337 | 0.288 | 0.161 | 0.225 | **0.016** |

**S3 Table. *cox*1 gene.** K2P Distance Matrix with estimates of evolutionary divergence over sequence pairs between groups. Bold numbers are the of evolutionary divergence within groups.
